# Michigan State Board of Health

**Published:** 1881-02

**Authors:** 


### ARTICLE XI.

MICHIGAN STATE BOARD OF HEALTH. Reported for the CHICAGO MEDICAL JOURNAL AND EXAMINER.

At the regular quarterly meeting of this board held on Tuesday, January 11, 1881, at its office in Lansing, the following members were present: R. C. Kedzie, M.D., president, of Lansing; Hon. Le Roy Parker, of Flint; Rev. D. C. Jacokes, D. D., of Pontiac; John H. Kellogg, M.D., of Battle Creek, and Henry B. Baker, M.D., secretary.

### VENTILATION.

Rev. Dr. Jacokes, committee on ventilation, reported some experiments which showed that through registers of equal size, one at the top and the other at the bottom of the room, the velocity of the upper current of air outward was greater than at the lower register. When the ventilation was from the bottom only, the temperature of the room was higher than when the ventilation was from both top and bottom registers. These experiments, he claims, demonstrate that ventilation should be from the bottom in this climate in winter.

Dr. Kedzie reported the following experiment, which seems to show the same fact: He took a glass tube thirty inches long having a thermometer in the lower end. When the tube was closed and the upper end heated to 750° F., the thermometer rose but one degree in an hour; the lower end of the tube being opened and air being drawn from it through the tube, the same heat being applied at the upper end, raised the thermometer below, over 100° in one minute.

Dr. Kedzie stated that in conversation with the newly elected governor, he had seemed to appreciate the work done by this board, and, in his message to the legislature had recommended an additional appropriation of $2,000 for the use of the board.

### LAWS DESIGNED TO PREVENT ACCIDENTS.

Mr. Parker, committee on legislation in the interests of public health, reported progress in the careful study of the laws relating to punishment for carelessness causing accidents, such as the falling of the "grand stand" at Adrian, and said in his opinion the laws are stringent enough now, but the sentiment of the people does not hold a man guilty of murder through an act of negligence. There was no law, however, requiring expert inspection of public buildings constructed or in course of construction. Mr. Parker also reported on a proposed system of

### INSPECTION OF STEAMBOATS

and other sailing vessels on our many inland lakes and streams, at summer resorts, etc. He had prepared a bill providing for such State inspection, and he was requested to take measures to have the bill presented to the legislature.

### THE WORK OF THE OFFICE.

The secretary's quarterly report of work, mentioned the preparation of diagrams and other labor in preparing and printing the report of the board for 1880, and similar work on two volumes of vital statistics; the distribution of documents published by the board, and of blanks for return reports; and preparations for the sanitary convention to be held, under the auspices of the board; 553 communications have been written during the quarter.

### ADULTERATION OF SUGARS.

The secretary reported that he had collected samples of sugars and syrups from the leading dealers in the city, and had received from Prof. S. P. Sharles, of Boston, the result of his analyses, which showed that the sugars were mostly not adulterated, and but two out of ten of the syrups. It is due to the dealers to state that those found to be adulterated were so sold by them, namely, as "corn sugar" syrups, "glucose" syrups, etc.

### DIPHTHERIA.

Dr. Kedzie mentioned a horrible superstition prevalent in Russia, under which a wafer is put into the mouth of a child suffering with the disease, and then into the mouth of a well child, with the idea that it is a protection against the disease. As it is a communicable disease, it would be difficult to devise a more certain mode of spreading it.

### POISONOUS JELLY.

A sample of apple jelly was sent to the secretary with the statement that eating of the jelly had caused the sickness of a large family. Dr. Kedzie had analyzed it and found three grains of sulphate of zinc to each ounce of the jelly. It was probably in the form of malate of zinc, formed by the action of the acid of the fruit on the galvanized iron vessel in which it was boiled. If this was the fact, it illustrated the danger of using such vessels for such purposes.

### YELLOWS IN PEACHES.

Dr. Kedzie reported an examination of peaches affected with the yellow. They were of fine appearance, rather red, especially about the pit. The meat was watery and decomposed rapidly. Chemical analysis showed excess of water and deficiency of sugar and jelly-forming material. He read letters from some who thought eating the peaches was not injurious to the health, and from others who stated the facts of sickness in repeated instances, after the eating of such peaches.

### "HOG CHOLERA."

Dr. Baker made a report as special committee to study the relations between the prevalence of "hog cholera" and the public health. His report included a statement of his trip to the southwestern part of the State where the disease prevailed, and numerous letters from farmers, physicians, veterinarians; among the latter Prof. Law, Prof. Klebe and Drs. Detness and Salnon.

A letter from Dr. Jerome, of Saginaw, stated that he saw hogs suffering with the disease, who were unable to go up the inclined plane at the slaughter-houses in Chicago, killed and made into lard, and stamped with a fancy brand. In this same connection, Dr. Baker spoke of

LARD WHICH CAUSED SEVERE SICKNESS

in a family in Lansing. A sample of the lard had been microscopically examined by Dr. Detmers, of Chicago, who sent drawings of the organisms he found in it, stating that they were the same as he had found to be the contagious principle in "hog cholera," sometimes called "swine plague." He also read a letter from Dr. Marshall of Lansing, which said he had examined a a sample of the lard in which the "fried-cakes" (which caused the sickness) were cooked, and had found the same organisms to be present. Dr. Baker also read a part of a letter from Prof. Klebs, of Prague, Austria, relating to the same subject. Prof. Klebs has made a special study of such subjects, and claims to have found the organism which is the specific cause of typhoid fever. He does not think hog cholera to be the same as typhoid fever, but would like material with which he could carry on a comparative study.

A vote of thanks was extended to those citizens who had labored so hard to make the

SANITARY CONVENTION AT FLINT

a success. The convention will be held on Jan. 25th and 26th, 1881.

Dr. Baker stated that

CONTAGIOUS DISEASES PREVAIL MOST WHERE

it was noticable that the local authorities paid little or no attention to the laws requiring the appointment of a health officer, and communication with this board.

The board adjourned to meet at Flint, January 25, 1881.

